# Synthesis, biological and electrochemical evaluation of glycidyl esters of phosphorus acids as potential anticancer drugs

**DOI:** 10.3762/bjoc.21.148

**Published:** 2025-09-15

**Authors:** Almaz A Zagidullin, Emil R Bulatov, Mikhail N Khrizanforov, Damir R Davletshin, Elvina M Gilyazova, Ivan A Strelkov, Vasily A Miluykov

**Affiliations:** 1 Arbuzov Institute of Organic and Physical Chemistry, FRC Kazan Scientific Center of Russian Academy of Sciences, Arbuzov Street 8, Kazan, 420088, Russia; 2 Institute of Fundamental Medicine and Biology, Kazan Federal University, Kremlyovskaya Street 18, Kazan, 420008, Russia; 3 Zelinsky Institute of Organic Chemistry, Russian Academy of Sciences, Leninsky prospekt 47, Moscow, 119991, Russia

**Keywords:** alkylating agent, glycidyl ester, electrochemical evaluation, phosphorus-containing drug

## Abstract

Organophosphorus compounds are important in synthetic organic chemistry and pharmaceutical applications due to their diverse biological activities. In this study, we synthesized three novel glycidyl esters of phosphorus acids **1**–**3** via the condensation of chlorophosphine oxides or phosphorus oxychloride with glycidol in the presence of a base, obtaining products with high purity and moderate to excellent yields. Their cytotoxic potential was evaluated using the MTT assay on human fibroblasts (HSF), prostate cancer (PC-3), and breast cancer (MCF7) cell lines, revealing moderate preferential cytotoxicity toward cancer cells, particularly in the case of MCF7. Additionally, linear sweep voltammetry (LSV) studies on human serum albumin (HSA) were conducted to investigate their alkylating properties. The electrochemical results suggest that these compounds effectively modify albumin, highlighting their potential as reactive anticancer agents. These findings provide important insights into the synthesis, cytotoxic activity, and biochemical reactivity of glycidyl esters of phosphorus acids, underscoring their potential as lead structures for further development in anticancer drug discovery and pharmaceutical research.

## Introduction

Phosphorus-containing drugs represent a crucial category of therapeutic agents, extensively utilized in clinical practice due to their diverse pharmacological properties and applications [1–4]. These compounds have garnered considerable attention from both pharmaceutical companies and researchers, reflecting their significance in drug development and therapeutic innovation [5–7]. The structural diversity of phosphorus-containing molecules, which includes phosphotriesters, phosphonates, phosphinates, phosphine oxides, and bisphosphonates, allows for tailored modifications that enhance selectivity, bioavailability, and reduce potential side effects [8–13]. This versatility makes them valuable not only as drugs but also as intermediates in synthetic organic chemistry, facilitating access to a wide array of molecular targets [14–16]. The importance of phosphorus-containing drugs extends beyond their therapeutic applications; they also play a pivotal role in addressing specific medical conditions such as chronic kidney disease (CKD) [17–18].

**Figure 1:**
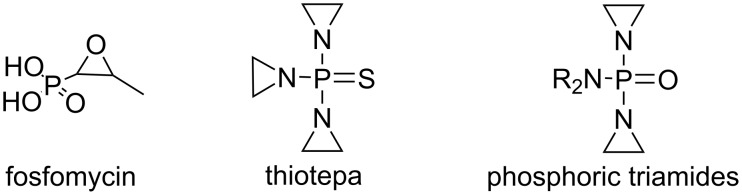
Structures of some pharmacological important phosphorus-containing molecules with oxirane or aziridine fragments.

The synthesis of organophosphorus compounds is a dynamic field of research, with numerous synthetic methodologies being explored to create novel phosphorus derivatives [19–21]. Recent studies have highlighted the increasing relevance of three-membered strained cycles containing phosphorus in various domains such as agrochemicals, synthetic chemistry, and medicine. This surge in interest has led to the development of innovative synthetic routes aimed at producing new members of these compounds [22]. For instance, fosfomycin [23] stands out as a broad-spectrum antibiotic currently employed in clinical settings, while thiotepa has been approved for treating several cancers, including gastrointestinal tumors and bladder cancer (Figure 1). Additionally, phosphoric triamides alkylating agents featuring aziridine rings are recognized for their role as nitrogen mustards in cancer therapy [24].

Although there are numerous examples in the chemical literature regarding the biological activity (including anticancer properties) of phosphoric esters, reports on biological studies of systems based on the P=O fragment and oxirane skeletons are less common. Nevertheless, systems containing both of the mentioned structural motifs are rarely encountered in the literature. In this paper, we report the synthesis, biological activity, and electrochemical evaluation of glycidyl esters of phosphorus acids.

## Results and Discussion

### Synthesis of glycidyl esters of phosphorus acids **1**–**3**

**Scheme 1:**
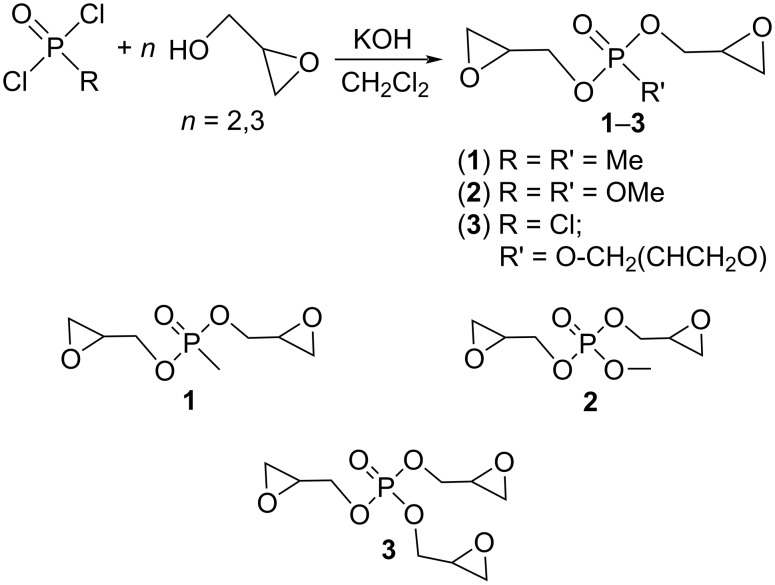
Synthesis of glycidyl esters of phosphorus acids **1**–**3**.

Glycidyl esters of phosphorus acids **1**–**3** were obtained by condensation of chlorophosphine oxides (methylphosphonic dichloride MeP(O)Cl_2_; methyl dichlorophosphate (MeO)P(O)Cl_2_) and phosphorus oxychloride P(O)Cl_3_ with racemic glycidol in CH_2_Cl_2_ in the presence of KOH as basic agent (Scheme 1). Further filtration and final distillation at low pressure leads to the products as thick liquids with good yields (44–67%) and purity.

The structures of glycidyl esters of phosphorus acids **1**–**3** were confirmed by ^31^P, ^1^H NMR, IR spectroscopy, and elemental analysis (see Experimental part for additional information). The ^31^P{^1^H} NMR spectrum of diglycidyl methylphosphonate (**1**) shows a singlet at +32 ppm; for diglycidyl methylphosphate (**2**) and triglycidyl phosphate (**3**) also a singlet in the region 0–1 ppm is observed, despite the presence of a chiral carbon atom in the oxirane fragment. In the ^1^H NMR spectra of esters **1**–**3** the characteristic signals of the oxirane fragment at 2.41–3.24 ppm and the POCH_2_- fragment at 3.66–4.36 ppm can be observed. The NMR data for the glycidyl esters of phosphorus acids **1**–**3** are comparable to those of related compounds.

### Biological activity of glycidyl esters of phosphorus acids

**Figure 2:**
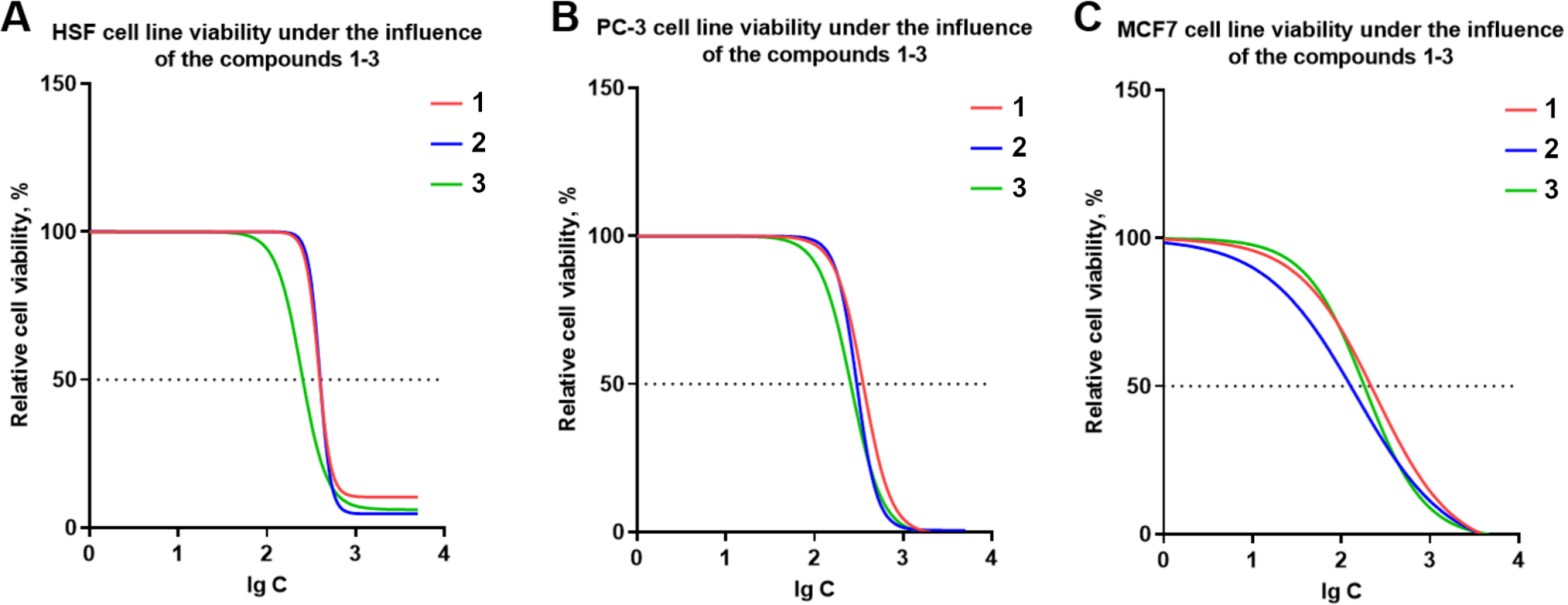
Dose–response curves for the cytotoxic effects of glycidyl esters of phosphorus acids **1**–**3** on human cell lines. (A) Human skin fibroblasts (HSF), (B) prostate cancer cells (PC-3), and (C) breast cancer cells (MCF7) were treated with diglycidyl methylphosphonate (**1**, red), diglycidyl methylphosphate (**2**, blue), and triglycidyl phosphate (**3**, green) for 48 hours. Cell viability was determined using the MTT assay. Data represent mean ± standard deviation (*n* = 3). The *y*-axis shows relative cell viability (%) compared to untreated control, and the *x*-axis indicates the logarithmic concentration of each compound. The dashed line marks the 50% viability threshold (IC_50_).

To evaluate the biological activity of diastereomeric mixtures of glycidyl esters of phosphorus acids **1**–**3**, their cytotoxic effects were assessed using the MTT assay on two tumor cell lines (PC-3 and MCF7) and one non-cancerous line (HSF). The assay measures the concentration of each compound required to inhibit cellular metabolic activity by 50% (IC_50_). All experiments were performed in biological triplicates, and standard deviations were calculated to assess statistical reliability. The results are summarized in Figure 2 and Table 1.

**Table 1:** IC_50_ values (μM ± SD) for glycidyl esters of phosphorus acids **1**–**3** in human skin fibroblasts (HSF), prostate cancer cells (PC-3), and breast cancer cells (MCF7).^a^

	IC50 (μM)		

Cell line	**1**	**2**	**3**

HSF	394 ± 28	398 ± 33	254 ± 19
PC-3	355 ± 25	300 ± 21	257 ± 20
MCF7	216 ± 16	128 ± 10	182 ± 14

^a^Values represent the mean ± standard deviation (*n* = 3) of biological replicates, determined by MTT assay after 48 hours of treatment. IC_50_ indicates the concentration required to reduce cell viability by 50%; lower values correspond to higher cytotoxic potency.

Diglycidyl methylphosphonate (**1**) reduced cell viability by 50% at concentrations of 394 ± 28 μM, 355 ± 25 μM, and 216 ± 16 μM for HSF, PC-3, and MCF7 cell lines, respectively. Similarly, diglycidyl methylphosphate (**2**) achieved 50% inhibition at concentrations of 398 ± 33 μM, 300 ± 21 μM, and 128 ± 10 μM. Triglycidyl phosphate (**3**) exhibited IC_50_ values of 254 ± 19 μM for HSF, 257 ± 20 μM for PC-3, and 182 ± 14 μM for MCF7 cells.

Among the tested compounds, triglycidyl phosphate (**3**) demonstrated the highest overall cytotoxicity against HSF and PC-3 cell lines, while diglycidyl methylphosphate (**2**) showed the greatest potency toward MCF7 breast cancer cells. Although the IC_50_ values for compounds **1** and **2** were somewhat higher in normal fibroblasts (HSF) compared to cancer cells, the differences were moderate (less than twofold). These results suggest a modest preferential cytotoxicity toward cancer cells, particularly in the case of compound **2** against MCF7, though further studies are needed to establish meaningful selectivity.

### Electrochemical studies

Alkylating agents are widely recognized for their ability to form covalent bonds with biological macromolecules (proteins, DNA). The literature discusses the interaction of small molecules with proteins, highlighting how linear sweep voltammetry (LSV) can be used to understand these interactions. The method provides insight into protein structures and functions using electrochemical methods that can also be applied to studies involving alkylating agents [25–26]. In this study, LSV was employed to investigate the interactions between human serum albumin (HSA) and the three prospective alkylating agents **1**–**3**. The motivation behind these experiments was to explore whether these compounds, which individually exhibit no appreciable redox activity in the potential window applied, can chemically modify (alkylate) serum albumin and thus suppress its characteristic oxidation peaks.

Human serum albumin was chosen as a model protein because of its well‐characterized structure and the presence of reactive sites that are known to be susceptible to alkylation. In standard aqueous media, the electrochemical oxidation of HSA can be observed via LSV as a broad wave, which is often attributed to the oxidation of amide and other amino acid side‐chain fragments. By tracking changes in this oxidation signal upon addition of an alkylating agent, we can infer whether the agent has effectively reacted with (and thus structurally altered) the protein.

**Figure 3:**
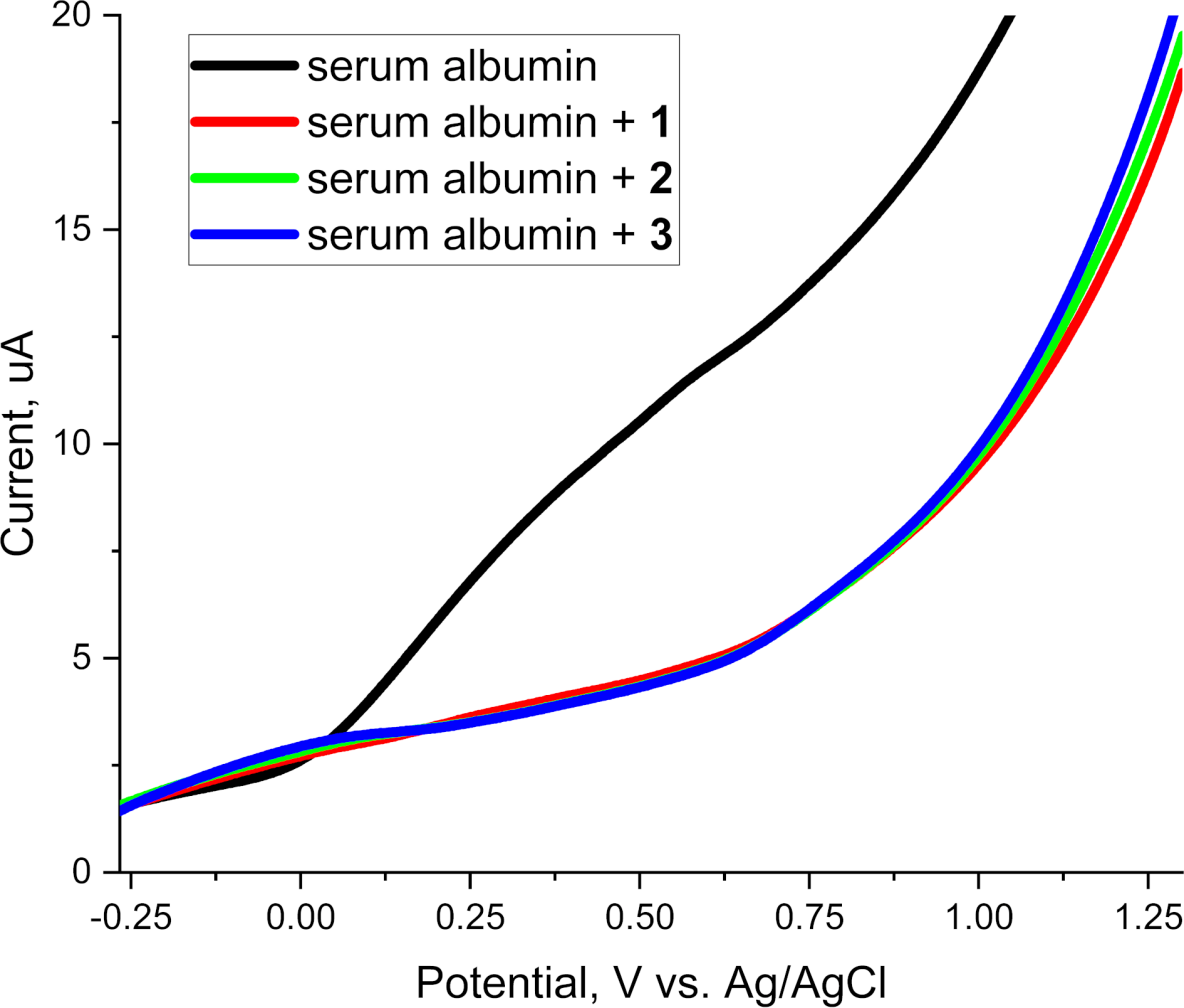
Linear sweep voltammograms of 1 × 10^−4^ М HSA (black) and HSA mixed with each of the three alkylating agents; **1** (red), **2** (green), and **3** (blue). Conditions: supporting electrolyte: 0.1 M Et_4_NBF_4_, working electrode: glassy carbon, scan rate: 0.1 V/s, pH 6.7.

As illustrated by the black trace in the LSV plot, pure HSA in aqueous medium shows a characteristic oxidation wave that begins to rise around +0.5 V and significantly increases up to +1.2 V vs Ag/AgCl (Figure 3). This wave is attributed to oxidation processes at peptide bonds or specific side chains (such as cysteine, methionine, tyrosine, serine, tryptophan residues), as well as the overall structure of the protein. The peak intensity and shape can vary depending on pH, ionic strength, and protein conformation. However, under our conditions, the HSA oxidation was consistent, well‐defined, and served as a clear baseline reference.

Subsequent to acquiring the control LSV of HSA, 10 µL of each alkylating agent was introduced separately into the albumin solution. As soon as the alkylating agent was added, the characteristic oxidation wave of the albumin nearly vanished or became drastically reduced. Control experiments confirmed that compounds **1**–**3** themselves exhibit no discernible redox activity in this potential range when tested in the absence of HSA. Consequently, any changes in the recorded voltammogram could be attributed to the interaction (alkylation) of albumin rather than to new electrochemical processes arising directly from the compounds.

**Figure 4:**
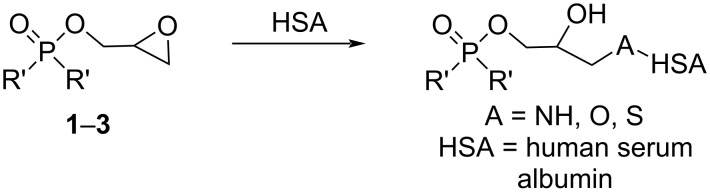
Chemical structure of alkylating fragment of **1**–**3** and the associated chemical pathway of its covalent attachment to HSA.

When these agents alkylate the HSA amino acid residues (particularly reactive sites like lysine, cysteine, serine NH_2_, SH, OH-side chains, and possibly other nucleophilic groups), the resulting covalent modification can disrupt the electroactive centers responsible for the protein’s oxidation peaks (Figure 4). In many alkylation scenarios, crosslinking or other structural rearrangements can render previously oxidizable moieties inaccessible or shift the protein’s conformational state. This suppresses or altogether eliminates the characteristic oxidation wave of HSA.

Based on established literature, the significant suppression or disappearance of the HSA oxidation peak upon addition of glycidyl esters **1**–**3** can be interpreted as evidence of covalent modification (alkylating) of nucleophilic sites on HSA, rather than non-specific binding or merely non-reactive association [27–29]. The observed disappearance of the albumin oxidation peak strongly suggests that all three investigated compounds can modify proteins under physiological conditions. Moreover, the fact that each agent was capable of this disruption aligns well with prior tests on the studied cell lines (PC-3, MCF-7, and HSF), where differences in IC_50_ values reflected the degree of alkylating potency and the selective toxicity toward cancer cells.

## Conclusion

In this study, we synthesized and comprehensively characterized a series of glycidyl esters of phosphorus acids **1**–**3**, evaluating their structural features, cytotoxic potential, and electrochemical behavior. The compounds were efficiently obtained via the condensation of chlorophosphine oxides and phosphorus oxychloride with glycidol, affording high-purity products in moderate to good yields. Cytotoxicity studies revealed that all three compounds possess antiproliferative activity against cancer cell lines (PC-3 and MCF7), with diglycidyl methylphosphate (**2**) demonstrating the highest potency toward MCF7 cells. While all compounds exhibited some level of toxicity toward non-cancerous HSF cells, their IC_50_ values in fibroblasts were generally higher than those observed in tumor cells, indicating a trend toward moderate preferential cytotoxicity. These findings suggest that the glycidyl phosphorus esters, particularly compound **2**, may serve as promising lead structures for further exploration as anticancer agents.

A key innovation in this work was the application of linear sweep voltammetry (LSV) to investigate the alkylating properties of the synthesized compounds. Unlike traditional biochemical assays, this electrochemical approach enabled real-time monitoring of protein modifications. The significant suppression of human serum albumin (HSA) oxidation peaks following exposure to compounds **1**–**3** strongly indicates their ability to covalently modify nucleophilic sites in proteins. This finding underscores the potential of LSV as a rapid and effective tool for assessing alkylating reactivity, with implications for future drug development.

Overall, this study offers meaningful insights into the synthesis, cytotoxic behavior, and biochemical reactivity of glycidyl esters of phosphorus acids. The results support their potential as reactive anticancer candidates and lay a foundation for future structure–activity relationship studies and further development in medicinal chemistry.

## Experimental

**General.** All reactions and manipulations were carried out under dry pure N_2_ in standard Schlenk apparatus. All solvents were distilled from sodium/benzophenone or phosphorus pentoxide and stored under nitrogen before use. The NMR spectra were recorded on a Bruker MSL-400 (^1^H 400 MHz, ^31^P 161.7 MHz, ^13^C 100.6 MHz). ^1^H and ^13^C NMR data are reported with reference to solvent resonances, and ^31^P NMR spectra were reported with respect to external 85% H_3_PO_4_ (0 ppm). All experiments were carried out using standard Bruker pulse programs. Infrared (IR) spectra were recorded on a Bruker Vector-22 spectrometer. The elemental analyses were carried out at the microanalysis laboratory of the Arbuzov Institute of Organic and Physical Chemistry, Russian Academy of Sciences.

**Cell cultivation.** Adherent cell lines HSF (human skin fibroblasts), MCF7 (breast adenocarcinoma), and PC-3 (prostate cancer) were maintained in Dulbecco’s Modified Eagle Medium (DMEM) supplemented with 5% fetal bovine serum (FBS), 1 mM ʟ-glutamine, and antibiotics (penicillin 5,000 U/mL and streptomycin 5,000 µg/mL). Cells were incubated at 37 °C in a humidified atmosphere with 5% CO_2_. For cytotoxicity assays, cells were seeded into 96-well flat-bottom plates at a density of 5 × 10^3^ cells per well and allowed to adhere for 24 hours under standard culture conditions.

**Preparation of compound solutions.** Stock solutions of diglycidyl methylphosphonate (**1**), diglycidyl methylphosphate (**2**), and triglycidyl phosphate (**3**) were prepared by dissolving the compounds in the culture medium to a final concentration of 25 mM. These stock solutions were stored and used for subsequent treatments.

**Cell treatment.** 24 hours after seeding, cells were treated with the test compounds at final concentrations of 25 μM, 50 μM, 100 μM, 250 μM, 500 μM, 750 μM, 1,000 μM, 2,500 μM, and 5,000 μM. Each concentration was tested in triplicate. Control wells received an equivalent volume of culture medium without compounds and served as untreated negative controls.

**Cytotoxicity analysis of compounds.** After 48 hours of treatment, MTT reagent was added to each well at a final concentration of 0.5 mg/mL. Plates were incubated for 3 hours at 37 °C in a CO_2_ incubator to allow for formazan crystal formation. Subsequently, 150 μL of dimethyl sulfoxide (DMSO) was added to each well to solubilize the formazan. Plates were shaken for 15 minutes, and absorbance was measured at 590 nm using an Infinite M200 microplate reader (Tecan, Switzerland). Cell viability was calculated relative to the untreated control (set at 100%). Data were processed and analyzed using GraphPad Prism 10 software.

**Electrochemistry.** Linear sweep voltammograms were recorded using a BASi Epsilon Eclipse potentiostat (USA). The device includes a measuring unit, a DellOptiplex 320 personal computer with Epsilon-EC-USB-V200 software. As supporting electrolyte 0.1 M Et_4_NBF_4_ was used. A glassy carbon electrode modified with carbon paste (surface area 1 mm^2^) served as the working electrode. Ag/AgCl (0.01 M KCl) was used as a reference electrode. A platinum wire was used as an auxiliary electrode. The scanning rate was 100 mV s^−1^. Measurements were carried out in a thermostatted electrochemical cell (volume 5 mL) in an inert gas atmosphere (N_2_). Between measurements or before recording the voltammetric wave, the aqueous solution was actively stirred with a magnetic stirrer in an atmosphere of constant inert gas flow.

**Starting materials.** Methylphosphonic dichloride MeP(O)Cl_2_ and methyl dichlorophosphate (MeO)P(O)Cl_2_) [30] were prepared according to literature procedures. Phosphorus oxychloride P(O)Cl_3_ and glycidol were purchased from suppliers and used without additional purification.

**Synthesis of diglycidyl methylphosphonate (1).** A 500 mL flask with 200 mL of dichloromethane, equipped with a mechanical stirrer, was cooled to −30 °C. Then, 2 equiv of glycidol (21.4 g, 0.289 mol) and 4.2 equiv of potassium hydroxide (34.0 g, 0.607 mol) were added to the flask. Methylphosphonic dichloride MeP(O)Cl_2_ (1 equiv, 19.2 g, 0.144 mol) was added dropwise to the mixture with constant stirring in 1 hour. The reaction mixture was additionally stirred at −25 to −30 °C for 2 h and precipitating for 12 h, while the temperature did not rise above 0 °C. After removal of the precipitate by filtration at 25 °C (filter consisted of layers of Celite, sodium sulfate and activated carbon), the filtrate was evaporated under reduced pressure to remove dichloromethane and excess glycidol. After vacuum distillation (*p* = 1·10^−3^ mbar, bp = 130–136 °C) the product diglycidyl methylphosphonate (**1**) was obtained as a thick liquid in 58% yield (17.5 g). ^1^H NMR (CDCl_3_, δ, ppm, *J*, Hz) 1.31 (d, ^2^*J*_PH_ = 17.8, 3H, Me), 2.41–2.43 (m, 2H, CH_2_-oxirane), 2.58–2.61 (m, 2H, CH_2_-oxirane), 2.96–2.98 (m, 2H, CH-oxirane), 3.66–3.69 (m, 2H, OCH_2_), 4.07–4.09 (m, 2H, OCH_2_); ^31^P{^1^H} NMR (CDCl_3_, δ, ppm, *J*, Hz) 32.04 (s); IR (liquid, cm^−1^): 762 (m, oxirane), 858 (m,oxirane), 927 (m, oxirane), 1019 (m), 1139 (w), 1166 (w, P=O), 1240 (m, oxirane), 1316 (m, R-P(O)OR), 1349 (s, P=O), 1425 (w), 1455 (m), 1647 (m), 2932 (m), 3004 (m), 3066 (w); Anal. calcd for C_7_H_13_PO_5_: C, 40.39; H, 6.30; O, 38.43; P, 14.88; found: C, 40.24; H, 6.52; P, 14.79.

**Synthesis of diglycidyl methylphosphate (2).** A 500 mL flask with 200 mL of dichloromethane, equipped with a mechanical stirrer, was cooled to −30 °C. Then, 2 equiv of glycidol (20.0 g, 0.27 mol) and 4.2 equiv of potassium hydroxide (31.8 g, 0.567 mol) were added to the flask. Methyl dichlorophosphate (MeO)P(O)Cl_2_ (1 equiv, 20.1 g, 0.135 mol) was added dropwise to the mixture with constant stirring in 1 hour. The reaction mixture was additionally stirred at −25 to −30 °C for 2 h and precipitating for 12 h, while the temperature did not rise above 0 °C. After removal of the precipitate by filtration at 25 °C (filter consisted of layers of Celite, sodium sulfate and activated carbon), the filtrate was evaporated under reduced pressure to remove dichloromethane and excess glycidol. After two vacuum distillations (*p* = 1·10^−3^ mbar, bp 113–116 °C) the product diglycidyl methylphosphate (**2**) was obtained as a thick liquid in 44% yield (13.5 g). ^1^H NMR (CDCl_3_, δ, ppm, *J*, Hz) 2.49–2.55 (m, 2H, CH_2_-oxirane), 2.67–2.72 (m, 2H, CH_2_-oxirane), 3.06–3.14 (m, 2H, CH-oxirane), 3.65 (d, ^3^*J*_PH_ = 11.4, 3H, Me), 3.75–3.84 (m, 2H, OCH_2_), 4.13–4.23 (m, 2H, OCH_2_); ^31^P{^1^H} NMR (CDCl_3_, δ, ppm, *J*, Hz) −0.1 (s); IR (liquid, cm^−1^): 598 (w), 763 (m, oxirane), 865 (m, oxirane), 921 (m, oxirane), 1021 (m, P(O)(OR)_2_), 1140 (w, P(O)(OR)_2_), 1168 (s, P(O)(OR)_2_), 1185 (w), 1261 (m), 1350 (m, P=O), 1430 (w), 1455 (m), 1644 (w), 2858 (w), 2960 (w), 3008 (w), 3066 (w); Anal. calcd for C_7_H_13_PO_6_: C, 37.51; H, 5.85; O, 42.83; P, 13.82; found: C, 37.50; H, 6.03; P, 13.97.

**Synthesis of triglycidyl phosphate (3).** Synthesis of **3** was carried out in a manner similar to [31], but without sodium sulfate as a drying agent. A 500 mL flask with 200 mL of dichloromethane, equipped with a mechanical stirrer, was cooled to −30 °C. Then, 3 equiv of glycidol (22.6 g, 0.305 mol) and 4.5 equiv of potassium hydroxide (25.6 g, 0.457 mol) were added to the flask. Phosphorus oxychloride P(O)Cl_3_ (1 equiv, 15.6 g, 0.102 mol) was added dropwise to the mixture with constant stirring in 1 hour. The reaction mixture was additionally stirred at −25 to −30 °C for 3 h and precipitating for 12 h, while the temperature did not rise above 10 °C. After removal of the precipitate by filtration at 25 °C (filter consisted of layers of Celite, sodium sulfate and activated carbon), the filtrate was evaporated under reduced pressure to remove dichloromethane and excess glycidol. The product triglycidyl phosphate (**3**) was obtained as a yellowish thick liquid in 67% yield (18.1 g). ^1^H NMR (CDCl_3_, δ, ppm, *J*, Hz) 2.56–2.65 (m, 3H, CH_2_-oxirane), 2.74–2.85 (m, 3H, CH_2_-oxirane), 3.14–3.24 (m, 3H, CH-oxirane), 3.84–3.95 (m, 3H, OCH_2_), 4.22–4.36 (m, 3H, OCH_2_); ^31^P{^1^H} NMR (CDCl_3_, δ, ppm, *J*, Hz) −1.2 (s); IR (liquid, cm^−1^): 599 (w), 700 (w), 763 (m, oxirane), 797 (m, oxirane), 869 (m, oxirane), 918 (m, oxirane), 1024 (m), 1139 (w, P=O), 1166 (s, P=O), 1259 (m), 1349 (m, P=O), 1429 (w), 1454 (m), 1483 (w), 1520 (w), 1634–1644 (m), 2614 (w), 2899 (w), 2953 (m), 3006 (m), 3065 (m); Anal. calcd for C_9_H_15_PO_7_: C, 40.61; H, 5.68; O, 42.07; P, 11.64; found: C, 40.85; H, 5.82; P, 11.97.

## Supporting Information

File 1 ^1^H, ^31^P NMR and IR spectra of compounds **1**–**3**.

## Data Availability

All data that supports the findings of this study is available in the published article and/or the supporting information of this article.
