# Supplementary material for: Synthesis, biological and electrochemical evaluation of glycidyl esters of phosphorus acids as potential anticancer drugs
Source: Beilstein J Org Chem. 2025 Sep 15;21:1909–16. doi: 10.3762/bjoc.21.148 (PMC12456076; doi:10.3762/bjoc.21.148)
Supplement: File 1 — 1H, 31P NMR and IR spectra of compounds 1–3. [file Beilstein_J_Org_Chem-21-1909-s001.pdf]

BEILSTEIN JOURNAL OF ORGANIC CHEMISTRY

Supporting Information

for

Synthesis, biological and electrochemical evaluation of glycidyl esters of phosphorus acids as potential anticancer drugs

Almaz A. Zagidullin, Emil R. Bulatov, Mikhail N. Khrizanforov, Damir R. Davletshin, Elvina M. Gilyazova, Ivan A. Strelkov and Vasily A. Miluykov

Beilstein J. Org. Chem. **2025,** *21,* 1909–1916. doi:10.3762/bjoc.21.148

^{1}H, ^{31}P NMR and IR spectra of compounds 1–3

License and Terms: This is a supporting information file under the terms of the Creative Commons Attribution License (https://creativecommons.org/licenses/by/4.0). Please note that the reuse, redistribution and reproduction in particular requires that the author(s) and source are credited and that individual graphics may be subject to special legal provisions.
The license is subject to the *Beilstein Journal of Organic Chemistry* terms and conditions: (https://www.beilstein-journals.org/bjoc/terms)

^{31}P, ^{1}H NMR, IR spectra for diglycidyl methylphosphonate (1).

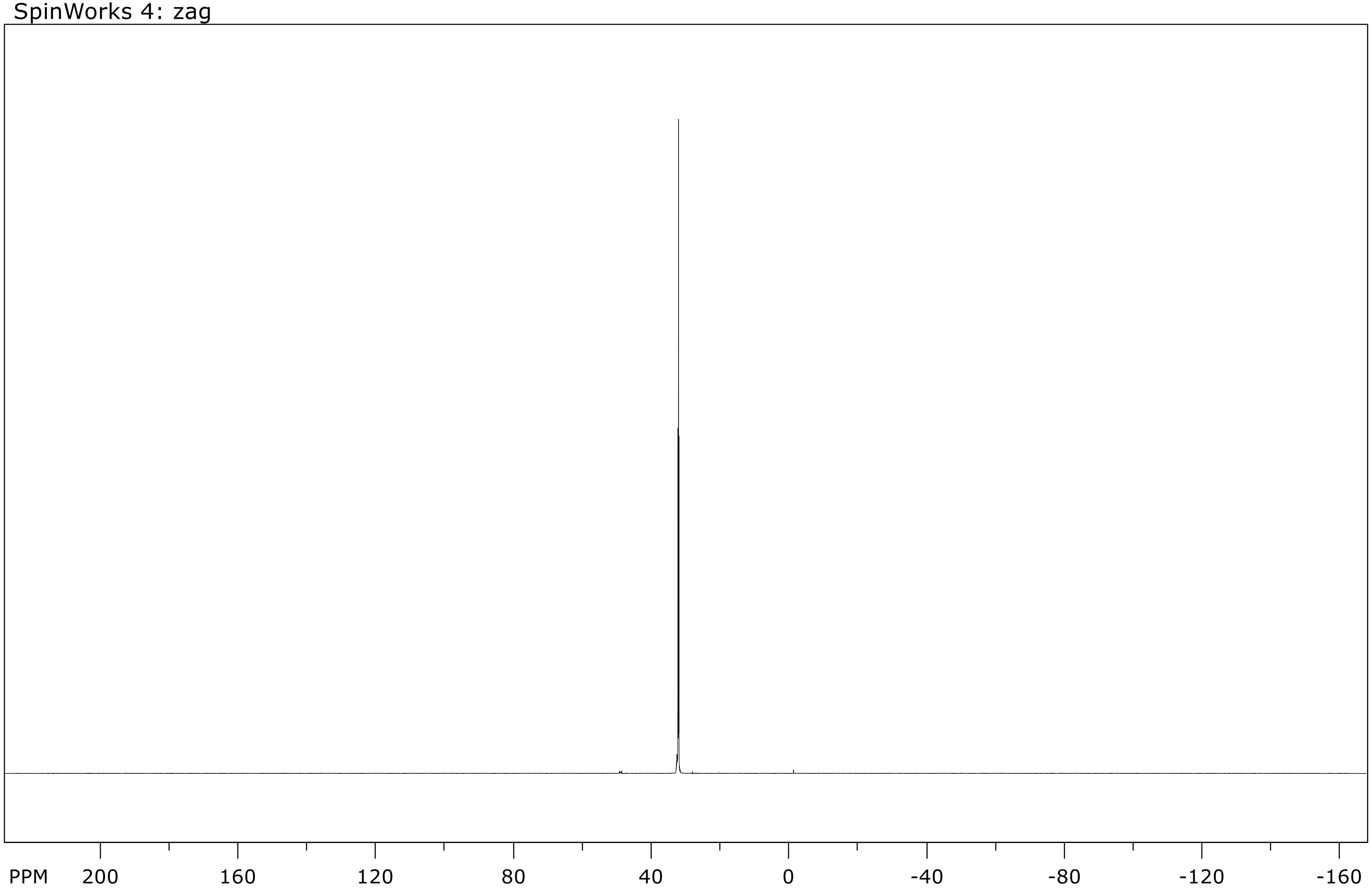

Figure S1. $^{31}P\{^{1}H\}$ NMR spectrum of **1** in $CDCl_3$.

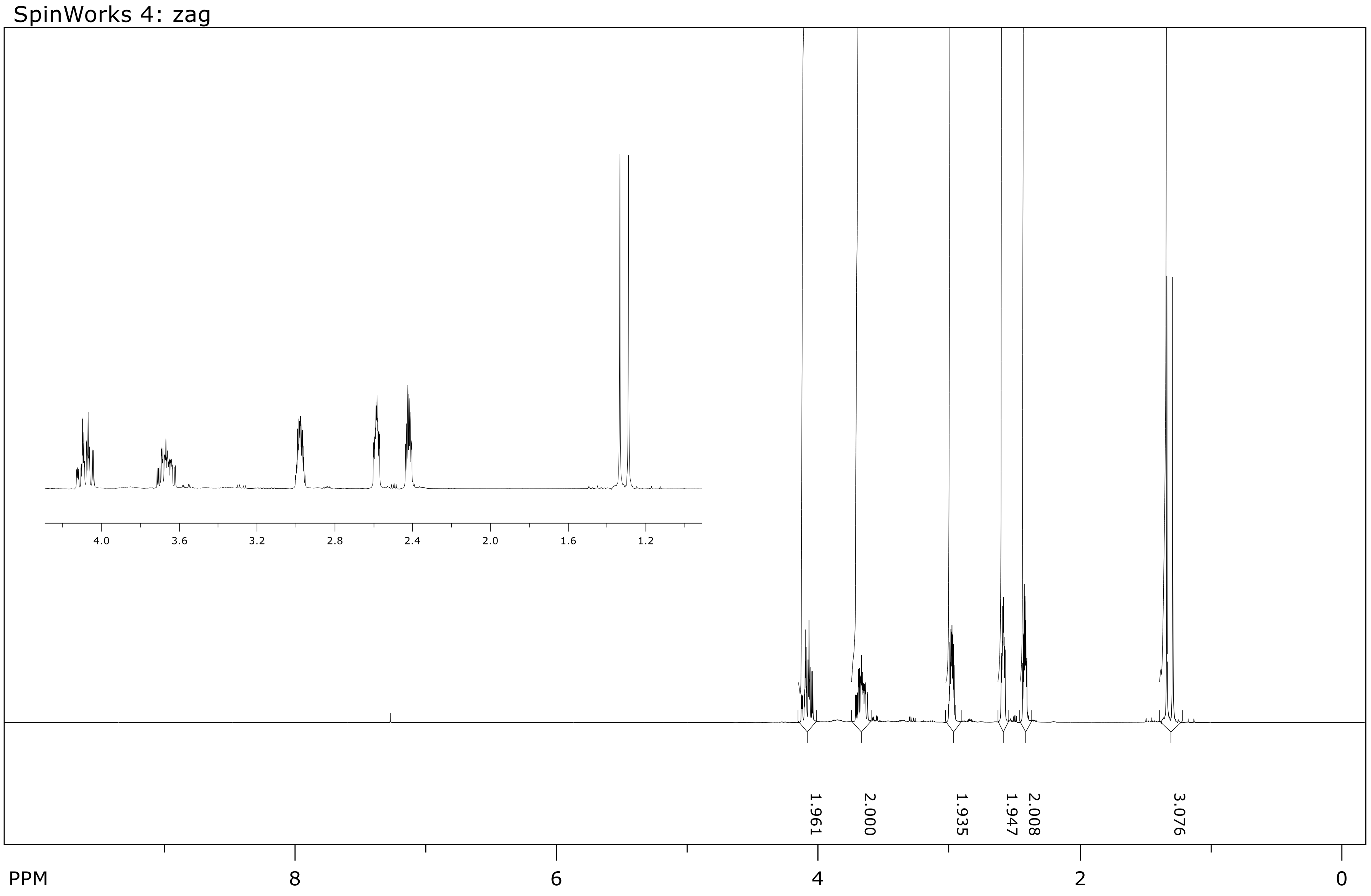

Figure S2. ^{1}H NMR spectrum of **1** in $CDCl_3$.

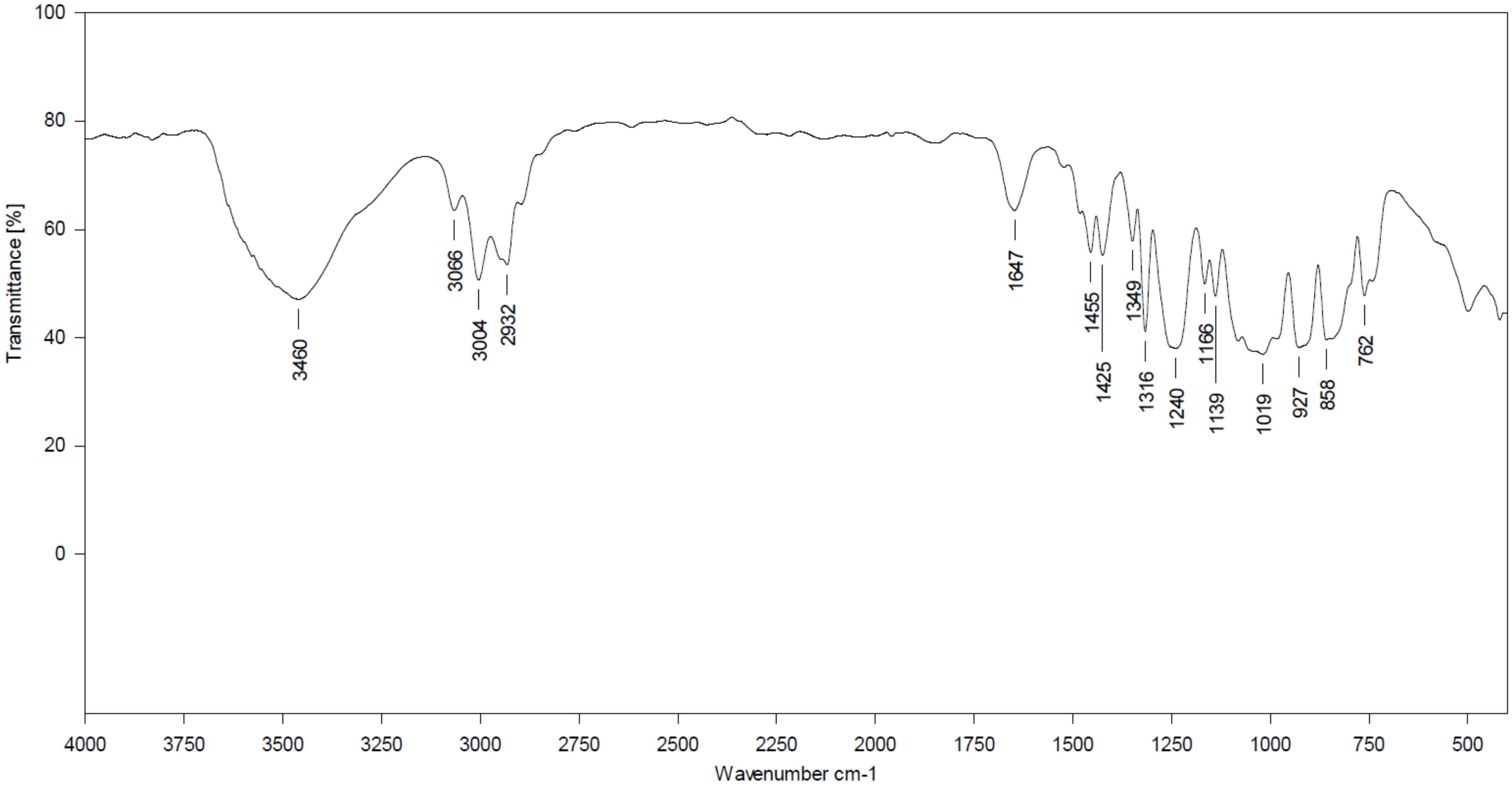

Figure S3. IR spectrum of **1** in $CDCl_3$.

^{31}P, ^{1}H NMR, IR spectra for diglycidyl methylphosphate (2).

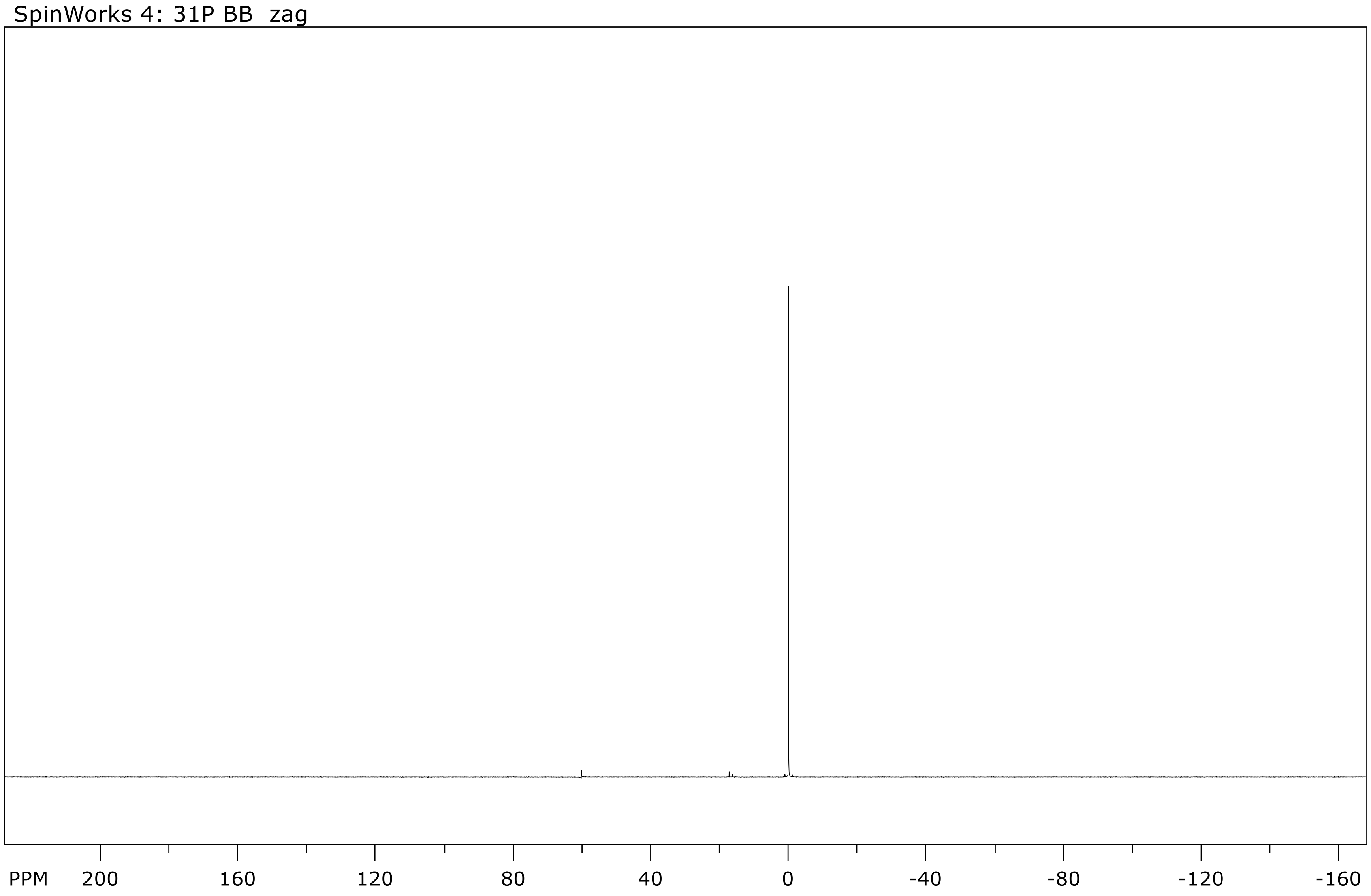

Figure S4. ^{31}P{^{1}H} NMR spectrum of **2** in $CDCl_3$.

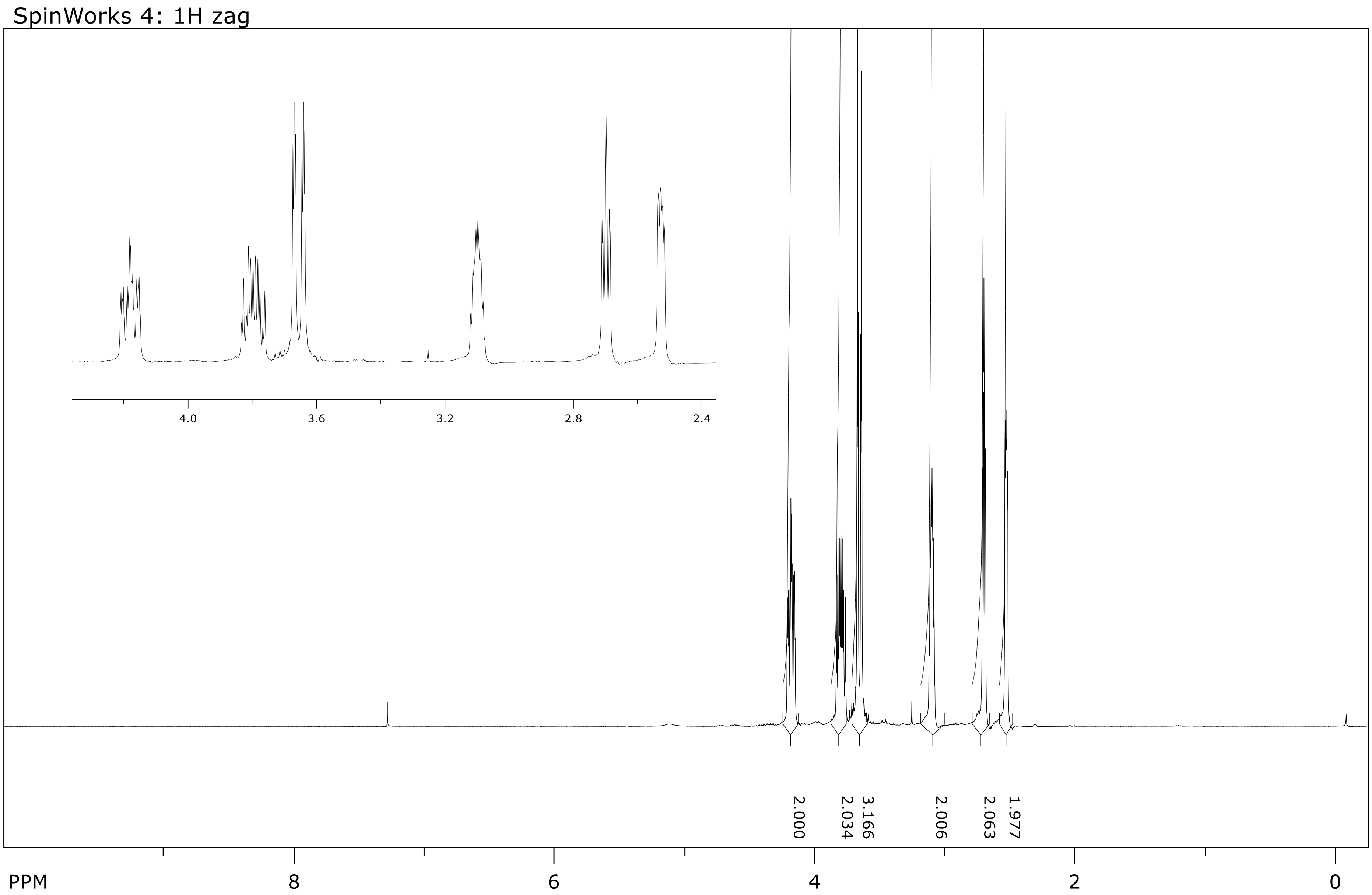

Figure S5. ^{1}H NMR spectrum of **2** in $CDCl_3$.

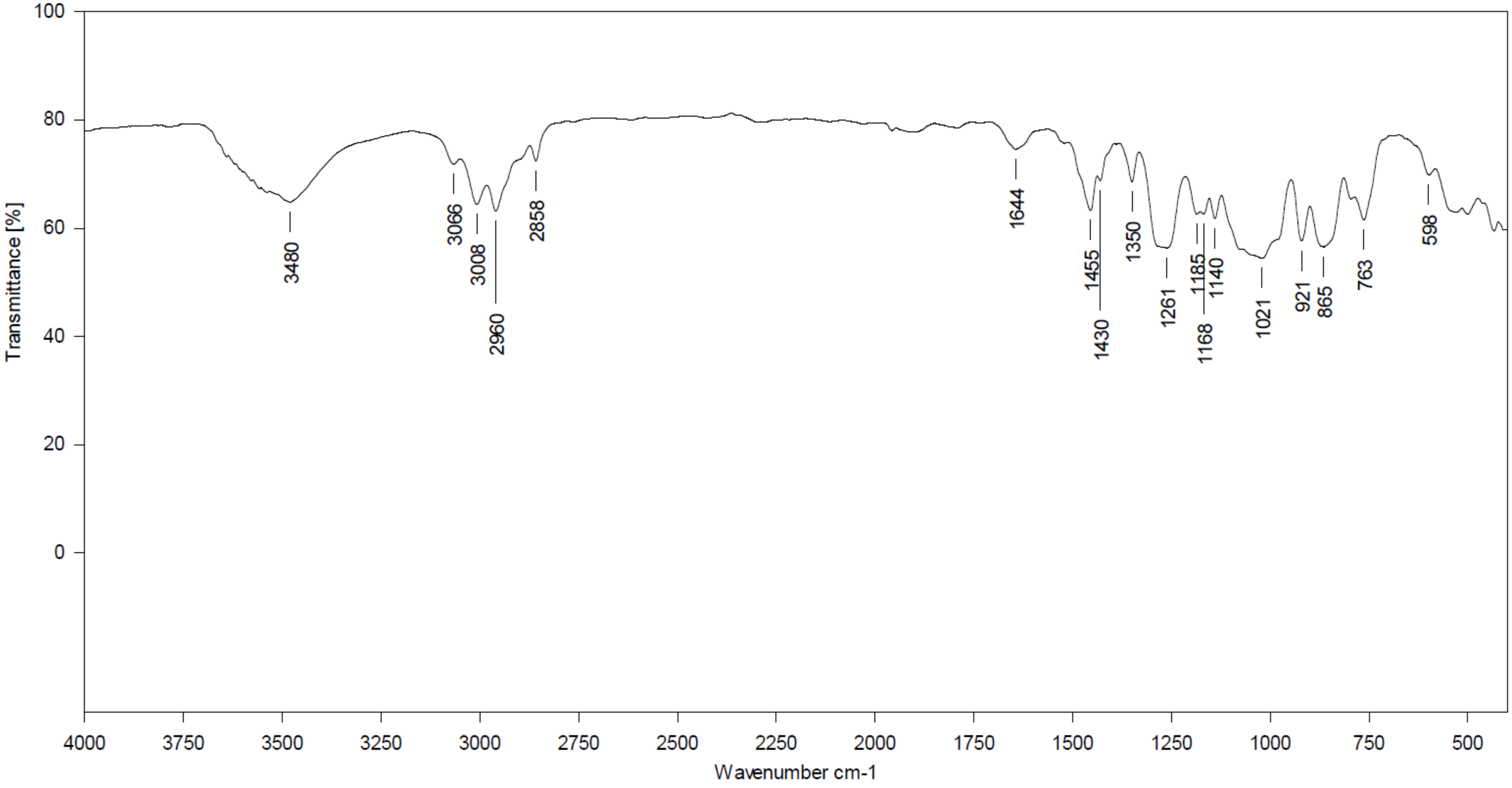

Figure S6. IR spectrum of **2**.

^{31}P, ^{1}H NMR, IR spectra for triglycidyl phosphate (3).

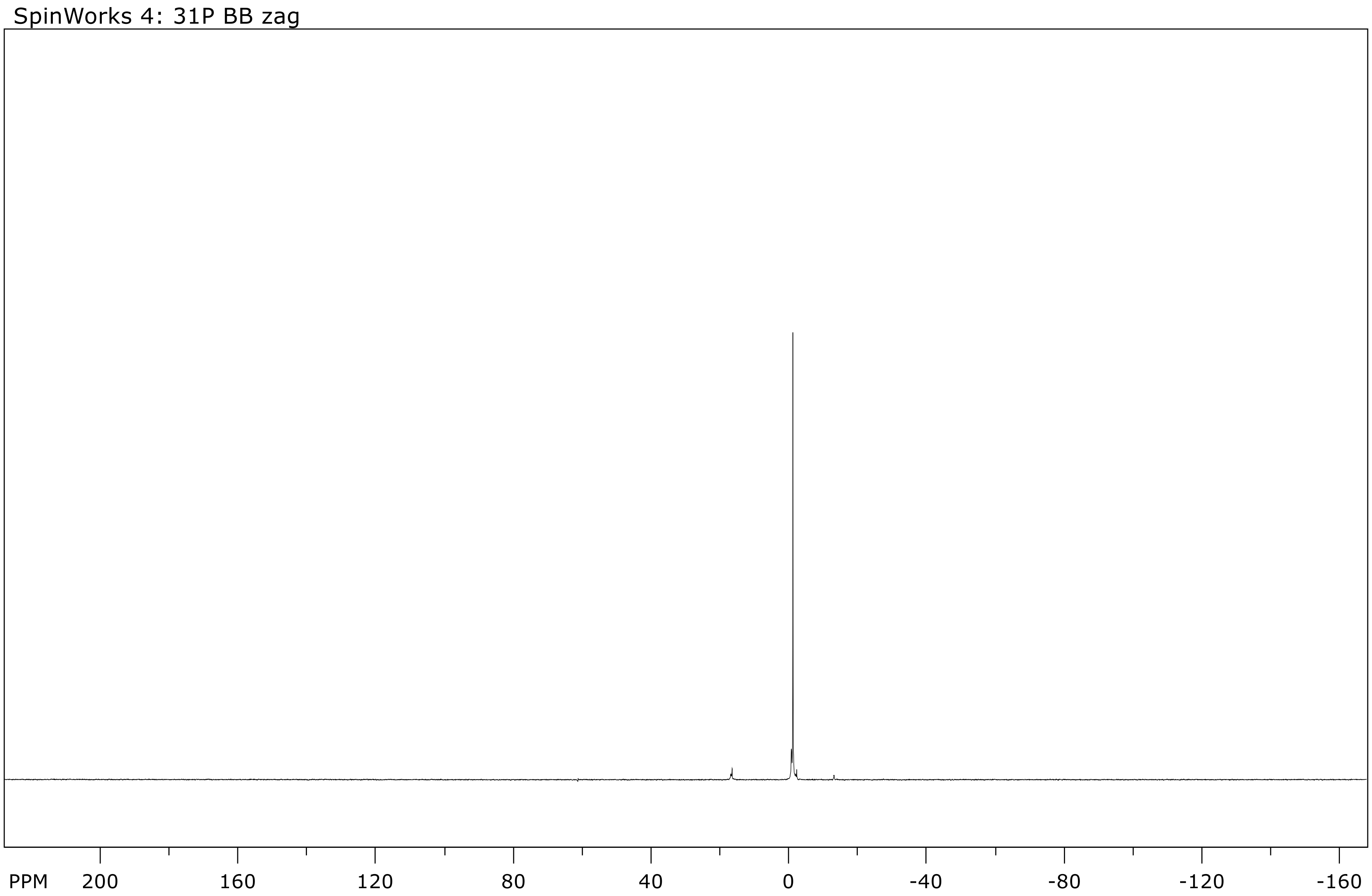

Figure S7. ^{31}P{^{1}H} NMR spectrum of **3** in $CDCl_3$.

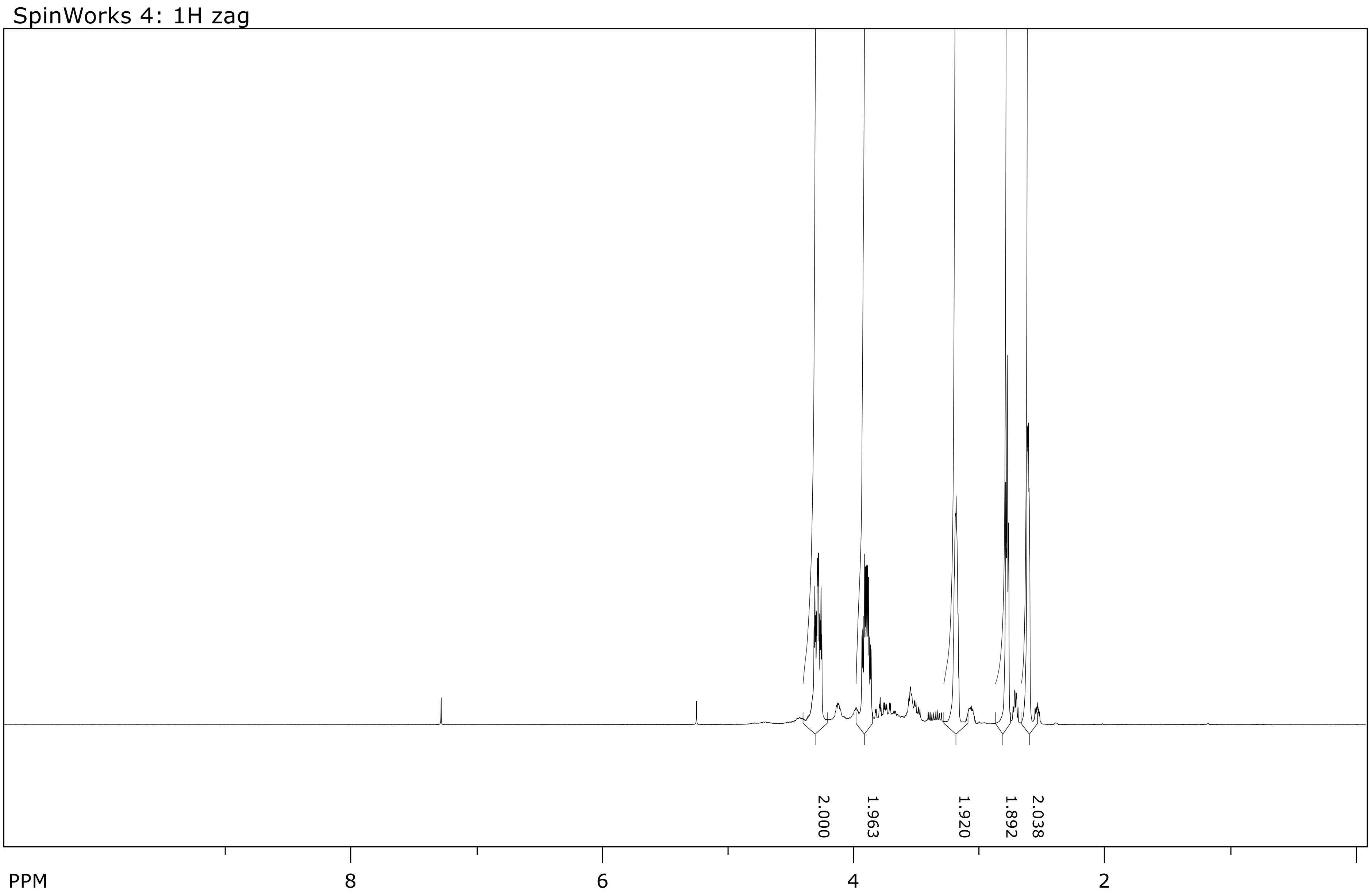

Figure S8. ^{1}H NMR spectrum of **3** in $CDCl_3$.

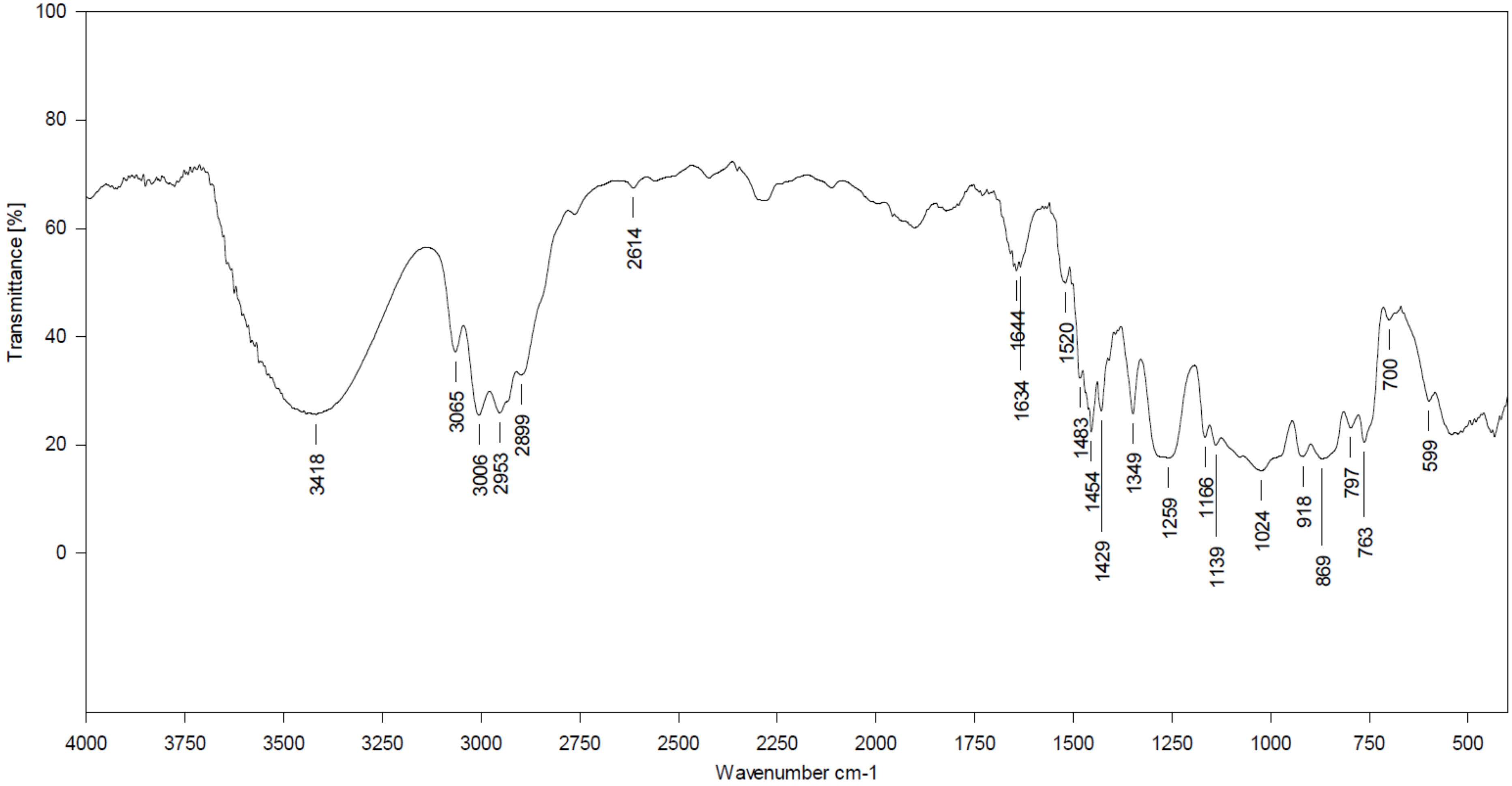

Figure S9. IR spectrum of **3**.
